# Supplementary material for: D-Dimer beyond Diagnosis of Pulmonary Embolism: Its Implication for Long-Term Prognosis in Cardio-Oncology Era
Source: J Pers Med. 2023 Jan 27;13(2):226. doi: 10.3390/jpm13020226 (PMC9962345; doi:10.3390/jpm13020226)
Supplement: Supplementary file 1 [file jpm-13-00226-s001.zip › jpm-2152620-supplementary.pdf]

Supplementary Table S1. The details of DOACs at discharge.

	malignancy		
	**No**	**inactive**	**active**
**Edoxaban**	46 (35%)	18 (51%)	50 (66%)
**Rivaroxaban**	26 (20%)	7 (20%)	10 (13%)
**Apixaban**	6 (5%)	2 (6%)	4 (5%)
**Dabigatran**	1 (0.7%)	0 (0%)	0 (0%)
**Warfarin**	39 (30%)	2 (6%)	3 (4%)
**None**	12 (9%)	6 (17%)	9 (12%)

$p < 0.001$ among the groups. DOAC, direct oral anticoagulant.
